# Visualization of DC-SIGN-Mediated Entry Pathway of Engineered Lentiviral Vectors in Target Cells

**DOI:** 10.1371/journal.pone.0067400

**Published:** 2013-06-28

**Authors:** Yarong Liu, April Tai, Kye-Il Joo, Pin Wang

**Affiliations:** 1 Mork Family Department of Chemical Engineering and Materials Science, University of Southern California, Los Angeles, California, United States of America; 2 Department of Biomedical Engineering, University of Southern California, Los Angeles, California, United States of America; 3 Department of Pharmacology and Pharmaceutical Sciences, University of Southern California, Los Angeles, California, United States of America; Institut National de la Santé et de la Recherche Médicale, France

## Abstract

Dendritic cells (DCs) are potent antigen-presenting cells and therefore have enormous potential as vaccine targets. We have previously developed an engineered lentiviral vector (LV) that is pseudotyped with a mutated Sindbis virus glycoprotein (SVGmu), which is capable of targeting DCs through Dendritic Cell-specific ICAM3-grabbing Nonintegrin (DC-SIGN), a receptor that is predominantly expressed by DCs. In this study, we aimed to elucidate the internalization and trafficking mechanisms of this viral vector system through direct visualization of GFP-Vpr-tagged viral particles in target DCs, which was further corroborated by drug inhibition and dominant-negative mutants of cellular proteins that regulate the endocytic traffic. We demonstrated that our engineered LVs enter the cell via receptor-mediated clathrin- and dynamin-dependent endocytosis. Microtubule networks were also involved in a productive infection. Viral vector fusion was low-pH-dependent and occurred in the early endosomal stage of the intracellular transport. Autophagy was also examined for its effect on transduction efficiency, and we observed that enhanced autophage activity reduced vector infectivity, while suppressed autophagy boosted transduction efficiency. This study shed some light on the internalization and trafficking mechanisms of DC-directed LVs and offers some strategies to further improve the efficiency of LV-mediated gene therapy.

## Introduction

Vaccines are incredibly potent tools in immunology, capable of greatly reducing, and even eradicating as in the case of smallpox, vaccine-preventable infections. Dendritic cells (DCs) are excellent targets for vaccine therapy because they are considered to be the most potent antigen-presenting cells of the immune system [1], [2], [3], [4]. Efforts have been made to modify DCs by loading them with antigens *ex vivo*, or by genetically altering them using liposomes, gene-gun, or transduction by viral vectors [1], [5], [6]. Genetic alterations are attractive because they allow for longer periods of antigen presentation, the ability to use both MHC I and II epitopes, and they can include genes that enhance DC function [7], [8]. Lentiviral vectors (LVs) have been considered as an attractive vehicle for gene therapy because they are able to transduce nondividing cells and permanently integrate into the target cell genome [9], [10], [11]. Moreover, these vectors can be efficiently pseudotyped with other viral glycoproteins to alter their tropism and allow them to target specific cell types [12], [13].

Previously, we reported a method to engineer LVs to target the desired cell type [14]. The engineering approach involved a mutant form of Sindbis virus glycoprotein, designated as SVGmu, which has lost its ability to bind to heparin sulfate structures, while retaining its specific binding to human dendritic cell-specific intercellular adhesion molecule-3-grabbing nonintegrin (DC-SIGN). DC SIGN is a C-type lectin-like receptor that is expressed predominantly on DCs. Although it was shown that the engineered LVs could specifically transduce DCs and further induce DC maturation *in vivo*, little is known about their entry mechanism and subsequent intracellular trafficking pathways in the targeted DCs. By studying the human DC-SIGN (hDC-SIGN)-mediated transduction pathway by SVGmu-pseudotyped LVs (LV-SVGmu), we can achieve a greater understanding of the underlying mechanisms of vector transduction in DCs, which can aid in the further design and development of DC-based vaccine strategies, as well as offer crucial insights for improving virus-mediated gene delivery.

Generally, enveloped viruses utilize receptor-mediated endocytosis for entry, after which the viruses travel through endocytic compartments and fuse with the endosomal membrane, resulting in the release the viral genome into the host cell [15], [16], [17], [18]. To study these processes in our engineered LVs, we utilized a direct visualization technique via confocal microscopy to monitor the interaction between fluorescent-tagged viral particles and cellular endocytic components in the target cells. Our results suggest that the LV-SVGmu virions enter the hDC-SIGN-expressing cell line through clathrin-mediated endocytosis, after which they travel intracellularly along microtubule networks. Fusion of the LV-SVGmu is pH-dependent, and for most virions, fusion occurs after 20 min of incubation at 37°C with the cells. At 20 min, most of the viral particles are localized in early endosomes, which suggests that fusion is early endosome-dependent. Autophagy was also shown to play a role in viral infection because drug treatments revealed that the infection rate decreases as autophagy activity increases.

## Materials and Methods

### Cell Lines and Antibodies

The 293T/hDC-SIGN cell line was generated previously in our lab [14]. Briefly, the 293T/hDC-SIGN cell line was generated by stable transduction of parental 293T cells with a VSVG-pseudotyped lentivector encoding a human DC-SIGN gene. 293T and 293T/hDC-SIGN cells were incubated at 37°C and 5% CO2 in Dulbecco’s modified Eagle’s medium (Mediatech Inc., Manassas, VA, USA) with 10% fetal bovine serum (Sigma, St Louis, MO), 2 mM L-glutamine (Hyclone, Logan, UT), 100 U/ml of penicillin and 100 ug/ml of streptomycin (Gibco-BRL). Mouse monoclonoal antibody for EEA1, rabbit polyclonal antibody for CI-MPR, lysosome-associated membrane protein 1 (Lamp-1), anti-LC3A/B, and Alexa 647-conjugated goat anti-rabbit immunoglobulin G (IgG) antibody were obtained from Abcam (Cambridge, MA). Texas red-labeled goat anti-mouse IgG was purchased from Molecular Probes (Carlsbad, CA). Anti-α-tubulin mAb was purchased from Sgima (St. Louis, MO). Cyto-D, nocodazole, bafilomycin A1, taxol, chlorpromazine, filipin, rapamycin, and 3-MA were obtained from Sigma (St. Louis, MO).

### Plasmids

GFP-Vpr was cloned previously in our lab [19]. DsRed-hDC-SIGN was cloned by PCR-amplifying hDC-SIGN with HindIII and EcoR1 cut sites. The PCR product was then cloned with these cut sites into the pDsRed-Monomer-C1 obtained from Clontech (Mountain View, CA).

### Virus Production

GFP-Vpr-labeled lentiviruses (FUW/SVGmu/GFP-Vpr) were made by transiently transfecting 293T cells with a standard calcium phosphate precipitation method. The cells were transfected at about 80–90% confluency in 6 cm culture dishes with 5 µg of the lentiviral backbone plasmid FUW, with 2.5 µg each of GFP-Vpr, pSVGmu, and the packaging plasmids pMDLg/pRRE and pRSV-Rev. Four hours post-transfection, the cells were washed with medium and incubated for 48 h, after which the supernatant was collected and filtered with a 0.45-µm pore size filter. The high titer lentiviruses used for confocal imaging were then concentrated by ultracentrifugation (Optima L-90 K ultracentrifuge, Beckman Coulter, Brea, CA) for 90 min at 82,700 g and resuspended in 100 µl of Hank’s balanced salt solution (Hyclone, Logan, UT). Concentrated viruses were filtered by a 0.45 µm pore size centrifuge tube filter (Costar, NY) before experiments were conducted.

### Viral Transduction

293T/hDC-SIGN cells (0.2×10^6^ per well) were plated in a 24-well culture dish and spin infected with LV-SVGmu encoding a reporter GFP gene (2 ml per well) at 2500 rpm and 30°C for 90 min (Sorval Legend centrifuge). The cells were then washed and cultured for 3 days before FACS analysis of GFP^+^ cells. For drug treatments, cells were incubated with the drugs cyto-D (20 µM), nocodazole (60 µM), bafilomycin A1 (25, 50, and 100 nM), chlorpromazine (30 nM), filipin (15 nM), 3 MA (5 mM), and rapamycin (1 µM) for 30 min at 37°C before spin infection, as described earlier. Drug concentration was maintained during spin infection and incubated with the cells for 60 min at 37°C after infection and before replacement with fresh D10 medium.

### Confocal Imaging

A Yokogawa spinning-disk confocal scanner system (Solamere Technology Group, Salt Lake City, UT) with a Nikon eclipse Ti-E microscope equipped with a 60×/1.49 Apo TIRF oil objective and a Cascade II: 512 EMCCD camera (Photometrics, Tucson, AZ, USA) was used to acquire the fluorescent images. An AOTF (acousto-optical tunable filter)-controlled laser-merge system (Solamere Technology Group Inc., Salt Lake City, UT) was used to provide power for each of the laser lines: 491 nm, 561 nm, and 640 nm solid state lasers (50 mW for each laser). Hela/hDC-SIGN or 293T/hDC-SIGN cells were seeded onto glass-bottom culture dishes (MatTek Corporation, Ashland, MA, USA) and grown at 37°C overnight. The cells were then rinsed with PBS, and the concentrated viruses were added and incubated with the cells for 30 min at 4°C to synchronize infection. The cells were then shifted to 37°C for different time periods, washed with PBS to remove unbound and uninternalized viruses, and fixed with 4% formaldehyde for 10 min.

### Microtubule-mediated Transport

Viruses on microtubule networks were visualized by incubating the cells with GFP-Vpr-labeled LV-SVGmu for 1 h at 37°C. The cells were then fixed with 4% formaldehyde and permeabilized with 0.1% Triton X-100. The microtubules were then stained with anti-α-tubulin mAb (Sigma, St. Louis, MO) and Texas red-conjugated anti-mouse secondary antibody.

### Imaging Virus Fusion and Transport through Endosomes

For fusion studies, concentrated viruses were incubated with 100 uM of DiD, a lipophilic dye (Molecular Probes) for 1 h at room temperature. GFP-Vpr and DiD were simultaneously excited with a 488 nm Argon and a 633 nm HeNe laser, respectively. All samples were scanned under the same magnification, laser intensity, brightness, gain, and pinhole size conditions. For virus tracking with endosomal markers, the cells were incubated with GFP-Vpr-labeled viruses for different time periods at 37°C and fixed. They were then permeabilized with 0.1% Triton X-100 and immunostained with EEA1 and CI-MPR for early and late endosome markers, respectively. Texas red-conjugated anti-mouse IgG and Alexa 647-conjugated goat anti-rabbit IgG were used as secondary antibodies. For the visualization of viral particles in lysosomes, permeabilized cells were immunostained with Lamp-1 before secondary antibody staining with the Texas red-conjugated anti-mouse IgG. To visualize viral particles in autophages, cells were stained with the antibody against LC3A/B, followed by secondary staining by anti-rabbit IgG.

### Dominant-negative Mutants for Endosomal Dependency

293T/hDC-SIGN cells were transiently transfected with either the dominant-negative mutant or the wild-type construct for Rab 5, Rab 7, or Dyn by a standard calcium phosphate precipitation method. 24 h post-transfection, the cells were seeded at 0.2×10^6^ cells per well in a 24-well culture dish and transduced with 2 ml of viral supernatant. The cells were analyzed for GFP expression by FACS at 3 days post-infection.

## Results

### DC-SIGN-mediated and Clathrin-dependent Virus Entry

**Figure 1:**
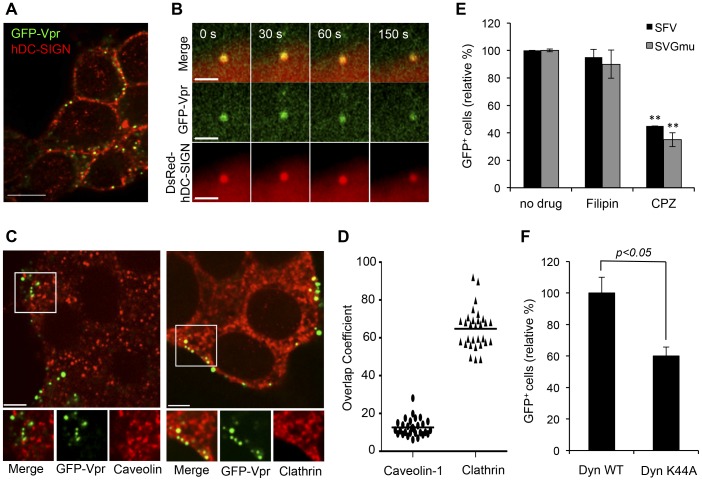
DC-SIGN-mediated and clathrin-dependent entry of LV-SVGmu. (A) Viruses binding to DC-SIGN-expressing cells. 293T/hDC-SIGN cells were incubated with GFP-Vpr-labeled LV-SVGmu particles (green) for 30 min at 4°C, fixed, and immunostained with anti-DC-SIGN antibody (red). (B) Real-time monitoring of virus internalization mediated by the DC-SIGN receptor. 293T cells seeded on a glass-bottom dish were transiently transfected with DsRed-DC-SIGN (red). At 24 h post-transfection, the cells were incubated with GFP-Vpr-labeled LV-SVGmu particles (green) for 30 min at 4°C and were then warmed to 37°C to initiate virus internalization. Confocal time-lapse images were then recorded. Selected frames of the real-time imaging are presented. (C) 293T/hDC-SIGN cells were incubated with GFP-Vpr-labeled LV-SVGmu viruses (green) for 30 min at 4°C to synchronize infection and were then shifted to 37°C for 5 min. After incubation, cells were fixed, permeabilized and immunostained with antibody against caveolin-1 (red) or clathrin (red). The boxed regions are enlarged in the bottom panels. Scale bar represents 5 µm. (D) Quantification of GFP-Vpr-labeled LV-SVGmu particles colocalized with clathrin signals. Overlap coefficents were calculated using Mander’s overlap coefficient in Nikon NIS-Elements software by viewing more than 40 cells. (E) The effect of inhibitory drugs on LV-SVGmu or LV-SFV transduction. 293T/hDC-SIGN cells were preincubated with chlorpromazine (CPZ, 30 nM) or filipin (15 nM) for 30 min at 37°C. Cells were then infected with LV-SVGmu or LV-SFV encoding a reporter GFP gene for 90 min in the presence of drugs. After an additional 3 h of incubation, the medium was replaced with fresh media, and the percentage of GFP-positive cells was analyzed by flow cytometry after 72 h. All of the data were then normalized based on the actual percentage of GFP-positive cells of the no drug treatment group of LV-SVGmu (35.2±0.028%) or LV-SFV (33.5±0.34%). All data are shown as the means of triplicate experiments. Asterisk indicates comparison to the no drug treatment group (*P<0.05, **P<0.01). (F) Functional involvement of dynamin in LV-SVGmu transduction. 293T/hDC-SIGN cells were transiently transfected with the wild-type or dominant-mutant form of dynamin I (K44) for 24 h, then infected with LV-SVGmu. The percentage of GFP-positive cells was analyzed by flow cytometry at 72 h post-infection. The data are presented as the mean values ± SD (n >3). The data were normalized based on the actual percentage of GFP-positive cells of Dyn-WT-expressing cells infected by LV-SVGmu (30.9±3.09%).

We sought out imaging methods to elucidate intracellular dynamics of interactions between engineered LVs and target cells and to interrogate the molecular mechanism of viral transduction. To visualize the intracellular trafficking of individual LV-SVGmu particles within target cells, LVs were labeled with green fluorescent protein (GFP) fused to the N-terminus of the human immunodeficiency virus (HIV) accessory protein viral protein R, designated as GFP-Vpr, which was described previously [19]. First, virus-cell binding was visualized using confocal microscopy to confirm DC-SIGN-mediated binding of LV-SVGmu to target cells. GFP-Vpr-labeled LV-SVGmu particles were incubated at 4°C with 293T cells stably expressing hDC-SIGN (293T/hDC-SIGN) used as the target cell line. As expected, significant GFP signals were detected on the surface of the cells, confirming that LV-SVGmu virions successfully bound to the DC-SIGN-expressing cell line. To further verify DC-SIGN-mediated virus entry and trafficking, viruses were incubated with 293T cells expressing DsRed-hDC-SIGN at 4°C for 10 min. The cells were then shifted to 37°C, and live cell imaging began using time-lapse confocal fluorescence microscopy. Selected images obtained from a time series were shown in Figure 1B. The LV-SVGmu particle (green) initially bound to hDC-SIGN, as evidenced by colocalization of GFP-Vpr and DsRed-hDC-SIGN signals. This colocalization was maintained for longer than 150 s, suggesting that the virus was trafficking along with the hDC-SIGN receptor within the target cell.

Clathrin- and caveolin-mediated pathways of endocytosis have been well-characterized as main routes of many viruses and were thus examined [20]. To investigate the role of clathrin- or caveolin-dependent endocytosis in the entry of LV-SVGmu, we visualized the individual LV-SVGmu particles and endocytic structures (clathrin or caveolin) in 293T/hDC-SIGN. As shown in Figure 1C, LV-SVGmu particles were detected in the clathrin structure at 5 min after incubation, while no viral particles showed significant colocalization with caveolin. The quantification of LV-SVGmu particles colocalized with caveolin-1 or clathrin structures by analyzing more than 40 cells statistically suggested that the clathrin-mediated pathway was involved in the entry of LV-SVGmu, as shown in Figure 1D. To further confirm the role of clathrin-dependent endocytosis in viral entry, drug treatments were used to inhibit these two pathways of viral entry. Specifically, chlorpromazine (CPZ), which prevents clathrin polymerization and obstructs internalization mediated by clathrin-coated vesicles, was used to block clathrin-mediated endocytosis [21], while filipin, a drug that depletes cholesterol to inhibit caveolin-dependent internalization [22], was used to block caveolin-mediated endocytosis. Semliki forest virus (SFV) known to enter cells through clathrin-mediated endocytosis was used as a positive control [20]. As shown in Figure 1E, CPZ (30 nM) markedly decreased the transduction of LV pseudotyped with the SFV glycoprotein (LV-SFV) and LV-SVGmu, both of which carried a GFP reporter gene, by over 55% (P<0.01) and 65% (P<0.01), respectively. However, no significant inhibitory effect of filipin (15 nM) was observed in either LV-SFV or LV-SVGmu. These results indicated that LV-SVGmu enters the cell through clathrin-dependent endocytosis and that caveolin might not be involved in early-stage viral entry.

In addition, it was reported that the large GTPase dynamin was required for the formation of clathrin-coated vesicles and was therefore another indicator of clathrin-dependency [23]. Thus, to investigate the functional involvement of dynamin in the entry of LV-SVGmu, a dominant-negative (DN) mutant construct (Dyn-K44A) was used to disable dynamin function [24]. Both dynamin wild-type (WT) and DN mutant constructs were used to transfect 293T/hDC-SIGN cells 24 h prior to transduction by LV-SVGmu encoding a GFP reporter gene. FACS analysis at 3 days post-transduction showed that the cells containing the DN construct exhibited a significantly reduced level of infection by LV-SVGmu, compared to cells transduced by the wild-type dynamin construct, as shown in Figure 1F. Taken together, these results demonstrated that SVGmu-pseudotyped LVs are internalized into cells through clathrin-mediated endocytosis in a dynamin-dependent manner.

### Microtubule-Mediated Transport of Engineered LVs

**Figure 2:**
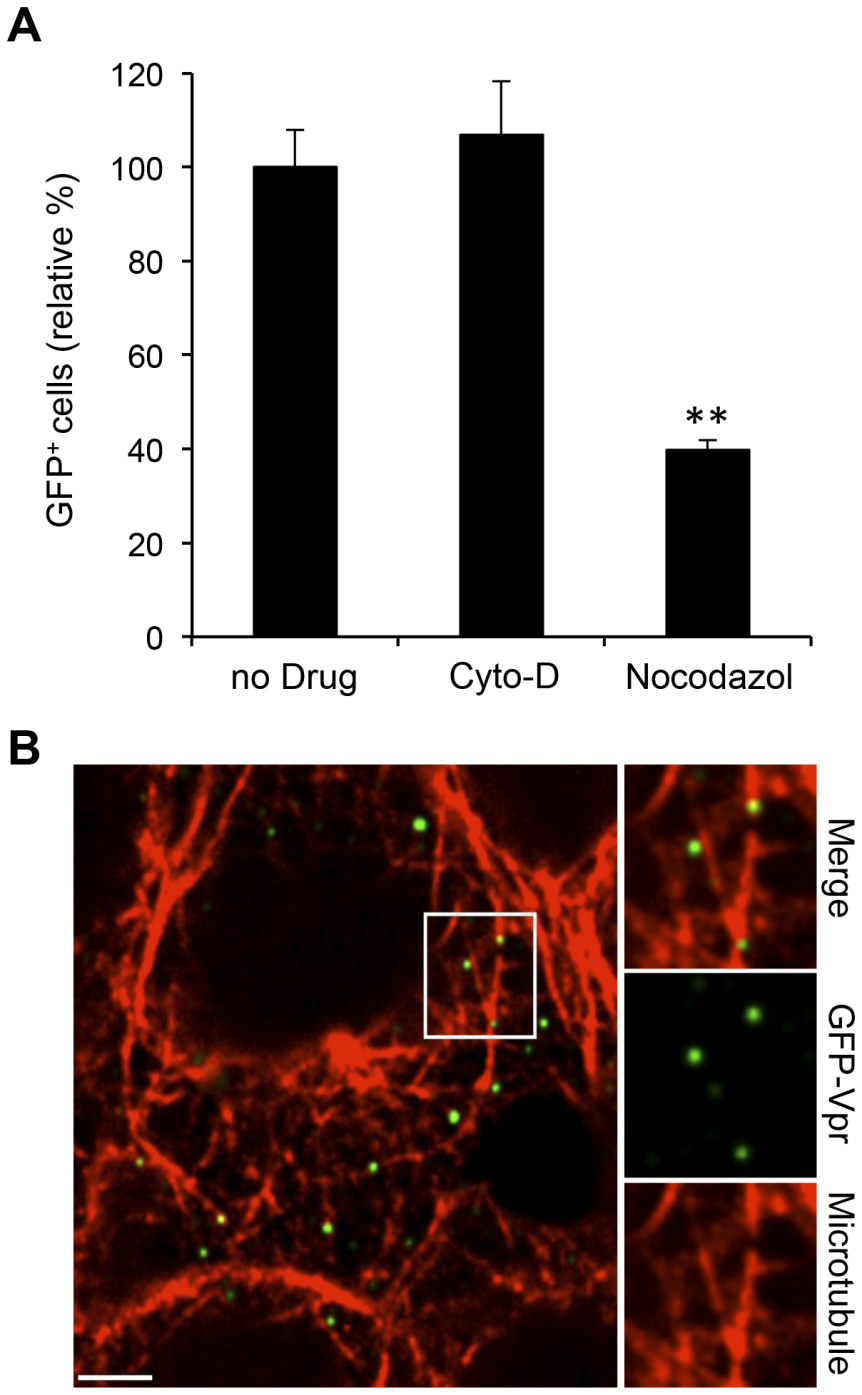
Microtubule network-mediated LV-SVGmu transport. (A) The effect of microtubule inhibitor on virus transduction. 293T/hDC-SIGN cells were preincubated with nocodazole (60 µM) or Cyto-D (20 µM) for 30 min and then infected with LV-SVGmu. The percentage of GFP-positive cells was analyzed by fluorescence-activated cell sorting (FACs). All of the data were then normalized based on the actual percentage of GFP-positive cells of the no drug treatment group of LV-SVGmu (44.1±3.48%). Error bars represent the standard deviation of the mean from triplicate experiments. (B) GFP-Vpr-labeled LV-SVGmu (green) was incubated with 293T/hDC-SIGN cells for 30 min at 37°C. Then the cells were fixed, permeabilized, and stained for microtubules (red) with rhodamine-conjugated phalloidin. The boxed regions are enlarged in right panels. Scale bar represents 5 µm.

Many viruses are known to rely on cellular transport using microtubule or actin networks of the cytoskeleton; for example, Herpes Simplex Virus 1 is known to utilize microtubules, while Epstein-Barr Virus requires actin for entry into B cells [25], [26]. Thus, we next aimed to determine whether actin filaments and/or microtubules were involved in viral transport. 293T/hDC-SIGN cells were incubated with the drugs cytochalasin D (Cyto-D) and nocodazole, which can inhibit actin polymerization and microtubule polymerization, respectively, before transduction with LV-SVGmu encoding a reporter GFP gene. FACS analysis of the GFP+ cells 3 days post-transduction revealed that the actin blocking by Cyto-D had no effect on viral infectivity, while microtubule blocking by nocodazole reduced infection levels to about 40% of the control (Figure 2A), suggesting the involvement of microtubules in intracellular trafficking of LV-SVGmu particles.

To further elucidate the functional involvement of microtubules in the viral transport within target cells, we conducted a colocalization experiment using α-tubulin-specific antibodies in 293T/hDC-SIGN cells. Visualization of the stained cells (Figure 2B) revealed that 53% of viral particles were detected on the microtubule structures (total ∼200 viral particles), confirming the results of the drug treatment. Taken together, these results suggest that a successful viral infection is dependent on the microtubule, but not actin, networks in the cell.

### Visualization of Viral Membrane Fusion

It is generally believed that fusion of virus envelope with the endosomal membranes is critical for the release of viral genome into cytosol before nuclear transport. To visualize the actual fusion event of LV-SVGmu within target cells, a double-labeling method with a lipophilic dye, 1,1′dioctadecyl-3,3,3′,3′- tetramethylindodicarbocyanine (DiD), and GFP-Vpr was used to label the viral membrane and core of LV-SVGmu, respectively [19]. The incorporation of DiD at high dye concentrations on viral membrane can lead to self-quenching of DiD fluorescence [19], [27], [28]. Viral fusion with endosomal membranes can be detected by DiD fluorescent dequenching that results from the dispersion of DiD dyes into endosomal membranes. It is also noteworthy that Vpr remains largely associated with the pre-integration complex of LVs after viral fusion [29], which enables us to further track uncoated viral cores that have been released to the cytosol from the fused endosomes by monitoring the GFP-Vpr signals.

**Figure 3:**
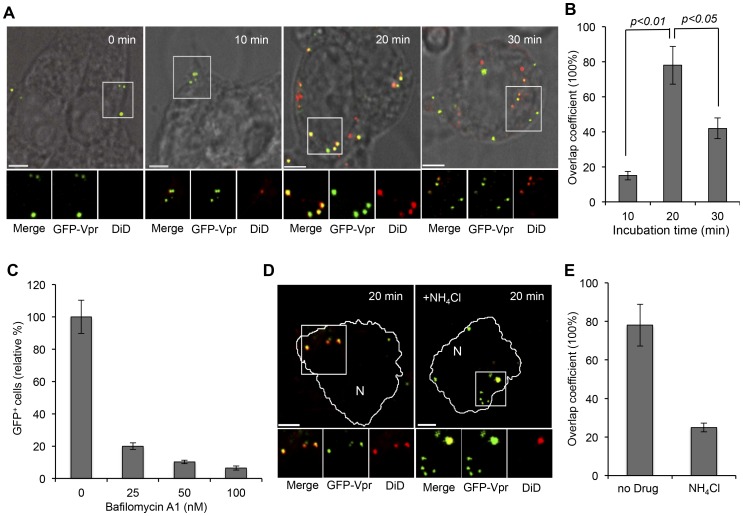
Low pH-mediated endosomal fusion of LV-SVGmu. (A) Visualization of virus-endosome fusion at different time points for LV-SVGmu. GFP-Vpr-labeled LV-SVGmu (green) were labeled with DiD (red) for 1 h at room temperature. Double-labeled viruses were added to 293T/hDC-SIGN cells for 30 min at 4°C to synchronize infection and were then shifted to 37°C for different incubation periods as indicated. The cells were then fixed and imaged. The boxed regions are magnified and shown below in individual panels. Yellow signals indicate viral particles fused with endosomes. Scale bar represents 5 µm. (B) Quantification of fused viral particles after 0, 10, 20 or 30 min of incubation. Overlap coefficients were calculated using Mander’s overlap coefficient in Nikon NIS-Elements software by viewing more than 40 cells at each time point. (C–E) Low pH-mediated endosomal fusion of LV-SVGmu. (C) The effect of the vacuolar proton ATPase inhibitor bafilomycin A1 on viral infection. 293T/hDC-SIGN cells were pretreated for 20 min with 0, 25, 50, and 100 nM bafilomycin A1. The cells were then infected with LV-SVGmu. After 90 min of infection and an additional 3 h of incubation in the presence of the drug, the cells were washed and resupplied with fresh media. The percentage of GFP-positive cells was analyzed by flow cytometry at 72 h post-infection. All of the data were then normalized based on the actual percentage of GFP-positive cells of the no drug treatment group of LV-SVGmu (37.8±3.89%). Error bars represent the standard deviation of the mean from triplicate experiments. (D) Imaging viral fusion events after NH_4_Cl treatment. Double-labeled viruses were added to NH_4_Cl -treated or untreated 293T/hDC-SIGN cells for 30 min at 4°C to synchronize infection and were then shifted to 37°C for 20 min. The cells were then fixed and imaged. The boxed regions are magnified and shown below in individual panels. Yellow signals indicate viral particles fused with endosomes. Scale bar represents 5 µm. (E) Quantification of fused viral particles with or without NH_4_Cl treatment after 20 min of incubation. Overlap coefficients were calculated using Mander’s overlap coefficient in Nikon NIS-Elements software by viewing more than 40 cells.

To investigate the endosomal fusion kinetics of LV-SVGmu in target cells, DiD/GFP-Vpr-double-labeled viruses were incubated with 293T/hDC-SIGN cells for various time periods (0, 10, 20, 30 min). As shown in Figure 3A, after 10 min of incubation, most viral particles were seen solely by the green signal, which indicated that the viral fusion had not yet occurred. However, after 20 min of incubation, many viral particles were shown to fuse with endosomes, as evidenced by significant colocalization (>76%) of LV-SVGmu particles (GFP-Vpr) and fusion signals (DiD) (Figure 3A and 3B). The images obtained after 30 min of incubation showed significantly lower colocalization of GFP-Vpr with DiD signals, suggesting that certain viral particles were presumably released into cytosol after the endosomal fusion. This quantitative colocalization analysis indicated that virus-endosome fusion of LV-SVGmu peaked at 20 min of incubation in DC-SIGN-expressing cells (Figure 3B).

It is also reported that viral fusion proteins undergo conformational change, which is induced by receptor-binding and/or pH change. To investigate the pH-dependency of viral membrane fusion of LV-SVGmu, drug treatment with bafilomycin A1, which specifically inhibits vacuolar proton ATPases [30], was utilized to block low pH-associated endosomal processes in DC-SIGN-expressing cells. The viral transduction efficiency was then measured in cells treated with different concentrations of the drug. The FACS analysis of GFP^+^ cells showed that the transduction of LV-SVGmu was remarkably inhibited by bafilomycin A1 treatment in a dose-dependent manner (Figure 3C). Similarly, the transduction of LV-SVGmu was also significantly decreased in cells treated with NH_4_Cl that can neutralize acidic endosomal compartments (data not shown). To reaffirm the low-pH dependency of the viral fusion, viral membrane fusion events were visualized in the presence of NH_4_Cl in cells. The viruses were tagged with DiD and GFP-Vpr and were incubated for 20 min with the cells. Imaging of the viruses revealed that only about 26% of the viruses were colocalized with the DiD dequenching signals in NH_4_Cl-treated cells, compared to the >70% colocalization observed when NH_4_Cl treatment was not used (Figure 3D and 3E), suggesting that viral fusion events were significantly inhibited by the NH_4_Cl treatment. These results indicate that LV-SVGmu requires a low pH-triggered endosomal fusion to complete transduction.

### Endosomal Trafficking of Engineered LVs

**Figure 4:**
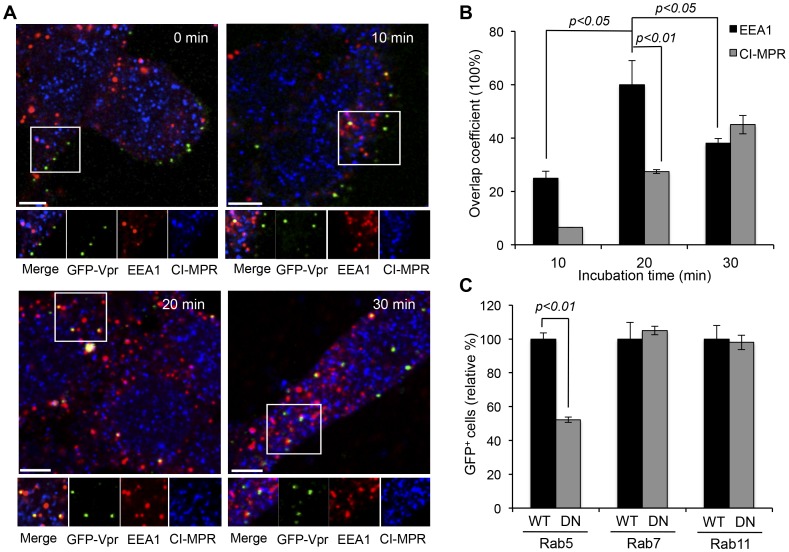
Functional involvement of various endosomes in LV-SVGmu transduction. (A) 293T/hDC-SIGN cells were incubated with GFP-Vpr-labeled LV-SVGmu viruses (green) for 30 min at 4°C to synchronize infection and were then shifted to 37°C for different indicated incubation periods. Subsequently, the cells were fixed, permeabilized and immunostained with antibodies against early endosome (EEA1, red) and late endosome (CI-MPR, blue). The boxed regions are enlarged in the bottom panels. Scale bar represents 5 µm. (B) Quantification of GFP-Vpr-labeled LV-SVGmu colocalized with EEA1^+^ or CI-MPR^+^ endosomes after 0, 10, 20 or 30 min of incubation. Overlap coefficients were calculated using Mander’s overlap coefficient in Nikon NIS-Elements software by viewing more than 40 cells at each time point. (C) 293T/hDC-SIGN cells were transiently transfected with wild-type or the dominant-negative mutant form of Rab5, Rab7 or Rab11. After 24 h, cells were infected with LV-SVGmu. The percentage of GFP-positive cells was analyzed by flow cytometry at 72 h post-infection. All of the data were then normalized based on the actual percentage of GFP-positive cells of wild-type Rab5-expressing cells (29.9±1.05%), wild-type Rab7-expressing cells (31.7±3.1%) or wild-type Rab11-expressing cells (28.6±2.26%), respectively. Error bars represent the standard deviation of the mean from triplicate experiments.

To elucidate the functional involvement of various endosomes in LV-SVGmu transduction of DC-SIGN-expressing cells, we visualized 293T/hDC-SIGN cells incubated with GFP-Vpr-labeled LV-SVGmu virions at different time points, which were then antibody-stained for early endosome antigen 1 (EEA1) [27], [31], [32] and cation-independent mannose-6-phosphate receptor (CI-MPR) [27], [32], [33] for the early and late endosomal markers, respectively. Images were taken at 0, 10, 20, and 30 min, correlating directly with the time points taken for the fusion study, and quantified (Figure 4A and 4B). At 0 min, none of the viral particles was colocalized with the endosomal markers, and most were only bound to the cell surfaces. At 10 min, more viruses had moved into the endosomes, with 25% of the viruses colocalized with the early endosomal marker, whereas no significant colocalization of viruses with the late endosomes was observed. At 20 min of incubation, which was also the time when most of the fusion events occurred, >60% of viruses were colocalized with the early endosomes, with about 27.4% in late endosomes. Correlation of this data with that of the fusion study leads us to believe that fusion occurs mostly at 20 min during the early endosome stage of endosomal transport for these hDC-SIGN-targeting viruses. In addition, at 30 min of incubation, 38% of the viruses were colocalized with the early endosomal marker, while 45% were colocalized with the late endosomal marker, indicating that the late endosomes might also be involved in the intracellular trafficking routes of LV-SVGmu.

For more quantitative data on the dependency of LV-SVGmu transduction on endosomal trafficking, dominant-negative mutant constructs of Rab proteins were employed to probe early (Rab5), late (Rab7) or recycling (Rab11) endosome function. Again, 293T/hDC-SIGN cells transfected with wild-type or the dominant-negative form of Rab5, Rab7 or Rab11 were transduced with LV-SVGmu carrying a reporter GFP gene, and GFP expression was analyzed by flow cytometry at 3 days post-transduction. As shown in Figure 4C, expression of dominant-negative Rab5 reduced the transduction of LV-SVGmu by ∼50%, as compared with the transduction of the wild-type Rab5-expressing cells, indicating that the viral trafficking to the early endosomes must be involved in the functional infection pathway. In contrast, LV-SVGmu transduction was not affected by the presence of dominant-negative Rab7 or Rab11 protein (P>0.05). The dominant mutant results suggested that viral transduction was indeed dependent primarily on the early endosomes and not much on either the late or recycling endosomes, consistent with the previous observation that the majority of SVGmu viral fusion occurs in the early endosomes. Although the colocalization study revealed a detectable level of colocalized viruses with the late endosomal marker (Figure 4A and 4B), perturbing Rab7 did not affect viral transduction, suggesting that late endosomes may not be directly involved in viral transduction. It is possible that this population of viruses detected in the late endosomes is carried via the transport process to lysosomes for degradation.

### Involvement of Autophagy in Viral Infection

Autophagy is the catabolic process by which cellular cytoplasm is enveloped into a double-membraned organelle, the autophagosome, and delivered to lysosomes for degradation [34]. This is required for the maintenance of cellular homeostasis, such as the degradation of protein aggregates from the cytoplasm and the removal of unwanted or damaged organelles, as well as for the removal of intracellular pathogens [35]. Several studies have examined the role of autophagy in viral infections, and it is believed that it has both anti- and pro-viral functions, aiding in the degradation of viruses or replication/release of viruses from infected cells, respectively, including, for example, vesicular stomatitis virus (VSV), coxsackievirus B3 (CVB3), and viral neurovirulence [36], [37], [38], [39]. Interestingly, it has been shown that autophagy plays several roles in HIV-1 biogenesis. In particular, early, nondegradative stages of autophagy enhance HIV-1 yields by promoting productive Gag processing. Furthermore, at the later stage of autophagy maturation, HIV-1 was subject to degradation. Importantly, however, this maturation process could be prevented by the HIV-1 protein Nef that acts an antiautophagic maturation factor through interaction with the autophagy regulatory factor, Beclin-1, thus protecting HIV-1 from autophagy-mediated degradation [40], [41]. In addition, autophagy appears to be involved in apoptosis of uninfected bystander CD4 T cells triggered by HIV-1 envelope glycoproteins expressed on HIV-1 infected cells [42], [43]. On the other hand, it has also been reported that autophagy plays no role in infection and production of the human rhinovirus (HRV) [44]. Although the multiple functions of the autophagy-lysosome network in virus production have also been increasingly explored, the role of autophagy in the viral infection process remains poorly understood. Thus, studying the function of autophagy during infection by LV-SVGmu might provide a better understanding of the essential steps involved for the successful transduction of LV-SVGmu.

**Figure 5:**
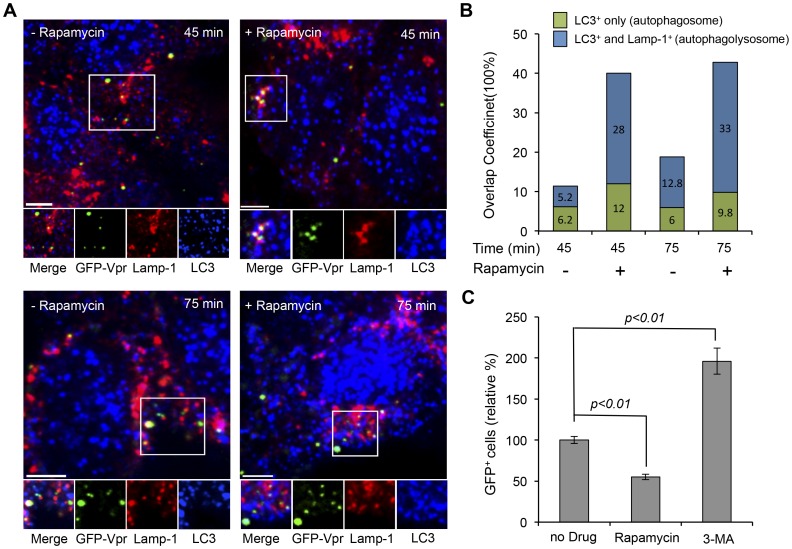
The role of autophagy and lysosome network in intracellular trafficking of LV-SVGmu. (A) Imaging the colocalization of autophages and lysosomes with LV-SVGmu particles with or without rapamycin treatment. 293T/hDC-SIGN cells treated or untreated by rapamycin were incubated with GFP-Vpr-labeled LV-SVGmu (green) for 30 min at 4°C to synchronize infection and were then shifted to 37°C for different indicated incubation periods. Subsequently, the cells were fixed, permeabilized and immunostained with antibodies against lysosomes (Lamp-1, red) and autophagy (LC-3, blue). The boxed regions are enlarged in the bottom panels. Scale bar represents 5 µm. (B) Quantification data on the colocalization of viral particles with autophagy-lysosome double-positive structure or autophagy alone in cells after rapamycin treatment. Overlap coefficients were calculated using Mander’s overlap coefficient in Nikon NIS-Elements software by viewing more than 40 cells at each time point. (C) The effect of autophagy activity on LV-SVGmu transduction. 293T/hDC-SIGN cells were preincubated with 3-MA (5 mM) or rapamycin (1 µM) for 30 min at 37°C. Then cells were infected with LV-SVGmu carrying a reporter GFP gene for 90 min in the presence of drugs. After an additional 3 h of incubation, the medium was replaced with fresh media, and the percentage of GFP-positive cells was analyzed by flow cytometry after 72 h. All of the data were then normalized based on the actual percentage of GFP-positive cells of the no drug treatment group of LV-SVGmu (25.8±1.14%). All data are shown as the means of triplicate experiments.

To investigate the role of autophagy in the intracellular trafficking pathway of LV-SVGmu, we visualized the individual GFP-Vpr-labeled particles along with an autophagic marker (LC3) and the lysosome marker (Lamp-1) in 293T/hDC-SIGN after 45 min or 75 min of incubation. LC3-positive punctuated structures represent the autophagosomal compartments, and the fusion of autophagosomes with lysosomes to form autophagolysosomes was identified as LC3- and Lamp-1-double-positive organelles whose contents were degraded by acidic lysosomal hydrolases [35], [45]. As shown in the left panel of Figure 5A, after 45 min incubation, 11.4% of viral particles were observed in LC3-positive autophagosomes, and 5.2% of viral particles were detected in autophagolysosomes double-positive for both LC3 and Lamp-1. At 75 min incubation, a number of viral particles found in autophagolysosomes were significantly increased (12.8%), suggesting that a good portion of LV-SVGmu particles tended to accumulate in autophagolysosomes, which could result in virus degradation. This result implied that autophagy might be involved in the transduction of LV-SVGmu in DC-SIGN-expressing cells.

To further verify the role of autophagy in viral trafficking, 293T/hDC-SIGN cells were treated with rapamycin, a drug known to enhance autophagy activity in cells, and incubated with GFP-Vpr-labeled LV-SVGmu for different indicated time periods (45 min or 75 min). As shown in the right panel of Figure 5A, at both 45 min and 75 min, rapamycin treatment significantly augmented the amount of viruses colocalized with autophagosomes and autophagolysosomes. The quantification of viral particles colocalized with LC3-positive or LC3- and Lamp-1-double-positive structures in the absence or in the presence of rapamycin was shown in Figure 5B. This result showed that the increased activity of autophagy by rapamycin treatment enhanced viral accumulation in autophagolysosomes, thus resulting in viral degradation. The result of this experiment suggested that autophagy presumably exerts antiviral effects during the viral transduction of LV-SVGmu.

To further confirm the functional role of autophagy in viral transduction, 293T/hDC-SIGN cells were treated with rapamycin or 3-methylademine (3-MA) before incubation with LV-SVGmu to enhance or reduce autophagy activity. As shown in Figure 5C, inhibition of autophagy activity by 3-MA resulted in a significant increase in transduction efficiency compared to that of untreated cells (p<0.01), and furthermore, the enhancement of autophagy by rapamycin treatment significantly lowered viral infectivity (p<0.01). Taken together, these results demonstrated that autophagy negatively affects the transduction of LV-SVGmu, thus promoting a marked viral degradation mediated by autophagolysosomal organelles in cells.

## Discussion

Dendritic cell-based vaccines have been considered as a potent method to stimulate antigen-specific immunity, owing to the superior ability of DCs as powerful antigen-presenting cells in capturing and presenting antigens. LVs are attractive antigen carriers for DC-based vaccine regimens because they can facilitate the delivery of antigen genes to less proliferative cells, such as DCs. In addition, LVs can be pseudotyped by various engineered viral glycoproteins for directed transgene delivery. Previously, we developed a LV pseudotyped with a mutated SVG glycoprotein, which was capable of targeting DCs through an DC-specific protein, DC-SIGN [14]. It has been demonstrated that these SVGmu-pseudotyped LVs could specifically transduce DCs expressing DC-SIGN both *in vitro* and *in vivo*. However, little is known about the intracellular trafficking pathways of this engineered virus in target cells. Understanding the infection pathway and mechanisms can be helpful for further optimizing this vector platform to deliver this new form of DC-based immunization.

The clathrin-mediated endocytic route has been considered as the most common endocytic pathway taken by viruses, such as adeno-associated viruses, vesicular stomatis virus or influenza A viruses [46], [47], [48], [49]. Moreover, alphaviruses, and, in particular, Sindbis viruses and Semliki Forest Virus (SFV) have been known to enter cells through clathrin-mediated endocytosis [47]. It was also reported the clathrin-coated pits were involved in the DC-SIGN-mediated uptake of ligands [50], antigens and viruses [51], [52]. Therefore, we hypothesized that the DC-SIGN-mediated entry of LV-SVGmu could be clathrin-dependent. As expected, by using drug inhibition, dominant-negative mutant and colocalization experiments, we demonstrated that SVGmu-pseudotyped LVs are internalized into cells via clathrin-coated pits in a dynamin-dependent manner.

It has been generally believed that endocytic clathrin-coated vesicles subsequently deliver their contents to the early endosome [53]. Through colocalization studies using endosomal markers, which were further confirmed by studies using dominant-negative mutants of Rab constructs, we showed that the early endosomes are required for the productive transduction pathway of LV-SVGmu. Some detectable colocalization of viruses with the late endosomes was observed, but since viral infection was not affected by the dominant-negative mutant of the late endosomes, they may not be involved in the infection pathway of LV-SVGmu. It is generally believed that late endosomes subsequently progress to lysosomes where viruses are degraded by proteases and hydrolases [54]. Thus, the subpopulation of SVGmu viruses trafficking though the late endosomes may further undergo degradation in lysosomes.

It has been reported that enveloped viruses respond to the pH drop in the acidic endosomal environment by undergoing conformational changes that lead to fusion [55]. For example, SFV has been known to fuse after arriving at the early endosomes [56], while influenza viruses are thought to be trafficked to the late endosomes where fusion occurs [54]. By tracking the fusion events of double-labeled LV-SVGmu viruses at various time points, we observed that most viruses undergo fusion at 20 min of incubation at 37°C, which also happens to be the peak time of colocalization of the early endosomal marker with viruses. This correlation suggests that the majority of LV-SVGmu fusion occurs in the early endosomes, a finding further confirmed by the reduced transduction of viruses in cells expressing the negative mutant form of Rab5. Although many viruses require a low-pH environment to trigger their conformational change for fusion, it has been reported that the pH thresholds that trigger viral membrane fusion are different for different viruses, which is generally determined by viral glycoproteins. For example, influenza viruses require a very low pH (∼pH 5.0) endosomal environment to trigger viral fusion, while Sindbis virus and SFV are known to have a relatively higher pH threshold for viral fusion (∼pH 6.0; close to the pH of early endosomes) [54], [57], [58], [59], which also supports our observation of LV-SVGmu fusion in the early endosomes.

In addition to fusion and endosomal escape, the degradation pathway of viruses is also a governing factor in the viral infectious process. Autophagy has been considered as a main pathway of viral degradation by fusing with lysosomes, subsequently maturing into autolysosomes, in which viruses are degraded [36]. For instance, it was shown that depletion of several autophagy proteins increased VSV infection [38]. Interestingly, many investigators have made inroads in exploring the multifunction of autophagy in the HIV-1 production process. Although autophagy is primarily considered to act as a degradative process, increasing evidence in HIV-1 reveals that it can assist in viral biosynthesis by promoting productive Gag processing in the early stages and preventing the degradative process in the later stages through the interaction of Nef with Beclin-1 [41], [42], [60]. Our results showed that autophagy plays an anti-viral role during LV-SVGmu transduction. By colocalization study using autophagy and lysosome markers and viral trafficking study using autophagy inducer (rapamycin) and inhibitor (3-MA), we demonstrated that the autophagy-lysosome network was involved in the degradative process of LV-SVGmu during infection.

This study demonstrates that autophagy activity could lower the transduction efficiency of DC-directed LVs, which, in turn, could lower the efficacy of vaccine delivery. It has been shown that autophagosomes may also trigger unwanted antivector immunity, which could negatively impact the desired vaccine-specific immunity [36], [61]. Thus, in conjunction with the vector-based platform described in the present study, it is important to consider the strategy to reduce autophage activity. Indeed, several autophagy inhibitors, such as 3-methyladenine (3-MA) and chloroquine (CQ), have been employed to inhibit autophagosome formation in several experimental settings [62], [63]. However, inhibition of autophage requires very high concentrations of 3-MA, which inevitably induce side effects and impact other cellular processes. In addition, CQ, which is a lysosomotropic agent, preferentially accumulating in the lysosomes of cells and raising intralysosomal pH, might block the pH-dependent fusion of LV-SVGmu and is perhaps not suited for enhancing LV-SVGmu-based vaccine delivery. Therefore, novel inhibitors of autophagy are highly desirable for this vectored vaccine system to increase the immunization effectiveness.
